# Development and validation of the body shape scale (BOSHAS) for assessing body shape perception in African populations

**DOI:** 10.1186/s12889-020-09654-w

**Published:** 2020-10-16

**Authors:** Emmanuel Cohen, Amadou Ndao, Jonathan Y. Bernard, Amadoune Gueye, Priscilla Duboz, Enguerran Macia, Gilles Boëtsch, Patrick Pasquet, Michelle Holdsworth, Philippe Jean-Luc Gradidge

**Affiliations:** 1 CNRS, UMR 7206 « Eco-anthropologie », Muséum National d’Histoire Naturelle, Paris, France; 2 CNRS, UMR 7178 « Institut Pluridisciplinaire Hubert CURIEN », Université de Strasbourg, Strasbourg, France; 3 MRC/Wits Developmental Pathways for Health Research Unit, Department of Paediatrics, Faculty of Health Sciences, University of the Witwatersrand, Johannesburg, South Africa; 4 CNRS, UMR 8177, IIAC-LAIOS, Ecole des Hautes Etudes en Sciences Sociales (EHESS), Paris, France; 5 Raum IG 551, Université de Francfort sur le Main, Institut Ethnologie, Frankfurt/Main, Germany; 6 Inserm, UMR 1153, Centre of research Epidemiology and Statistics Sorbonne Paris Cité (CRESS), Early Determinants of Children’s Health and Development (ORCHAD) team, Villejuif, France; 7 Paris Descartes University, Paris, France; 8 CNRS, UMI 3189 « Environnement, Santé, Société », Faculté de Médecine, UCAD, Dakar, Sénégal; 9 IRD, UMR 204 « Nutripass », Montpellier, France; 10 Centre for Exercise Science and Sports Medicine, Faculty of Health Sciences, University of the Witwatersrand, Johannesburg, South Africa

**Keywords:** Body image scale, Body shape perception, Validation, African ancestry, Africa

## Abstract

**Background:**

As a consequence of ‘Western’ acculturation, eating disorders and body image disturbances, such as fatness phobia and body dysmorphic disorders towards musculature and body shape, are emerging in Africa, with young people the most affected. It is therefore important to accurately assess perceptions of body shape. However, the existing body image assessment scales lack sufficient accuracy and validity testing to compare body shape perception across different African populations. The purpose of this study was to develop and validate the Body Shape Scale (BOSHAS) to evaluate body shape perceptions related body image disorders in African populations.

**Methods:**

To develop the BOSHAS, anthropometric measures of 80 Cameroonians and 81 Senegalese (both sexes included; 40.1% females overall) were taken for three body shape criteria: somatotype components, body mass index (BMI) and waist-to-hip ratio. Subjects were selected to cover a wide variability in body shape and were photographed in full face and profile positions. To validate the BOSHAS, the scale was administered twice (2 weeks apart) to 106 participants (aged 31.2 ± 12.6 years) to assess its reliability. In addition, a questionnaire measuring different aspects of body shape (e.g. musculature) was also administered (*n* = 597; aged 36.7 ± 15.6 years) to assess its convergent validity.

**Results:**

The BOSHAS includes two sex-specific subscales of 10 photographs each. Most participants were able to repeat their BOSHAS preference order. Test-retest reliability was also consistent in estimating Current Body Shape (CBS), Desired Body Shape and Ideal Body Shape for participants and their partners. CBS was correlated with BMI, and different BOSHAS indices were consistent with declarations obtained by questionnaire.

**Conclusions:**

The BOSHAS is the first sex-specific scale of real African models photographed in face and profile, including large body shape variability. The validation protocol showed good validity and reliability for evaluating body shape perceptions and dissatisfaction of Africans.

## Background

Body weight perceptions can be driven by body size norms portrayed in advertising, especially with the globalisation of contemporary consumer society [1]. This could result in body image disturbances and eating disorders in populations traditionally less acquainted with body image disorders related to ‘Western’ contexts [2–4]. Recent studies have shown that disordered dietary behaviors and poor perception of fatness are associated, in urban African adolescents and young adults living in Africa and African diaspora [5, 6]. Given the influence of the ‘Western’ lifestyle, obesity-related body image disorders are emerging in African populations, as observed in South Africa and Nigeria [5, 7]. Eating and body dysmorphic disorders are also emerging among underweight and normal-weight urban Black young people living in Africa and High Income Countries (HIC) [8–10]. Hence, investigating the ‘modern’ acculturation process around body perceptions, including the factors associated with valuing slimness, musculature and “sexy” shapes has started in African countries and African diaspora living in Europe [6, 11–14].

Although the literature highlights the importance of understanding changes in body shape perceptions in African populations to prevent the development of eating and body dysmorphic disorders [6, 15, 16], common methodological approaches to assess and understand body perceptions in African populations remain limited. To date, body shape perceptions have been assessed through a combination of qualitative and quantitative methods, including interviews, focus groups [17] and questionnaires [18], but these methods from discourse rather than visual do not allow the interviewees’ representations of the body to be accurately assessed. Figural stimuli representing different body shapes overcome this limitation [19, 20]. Drawings and silhouette scales are the usual figural stimuli, since they allow for the visual depiction of human body shapes across a spectrum, but they have limitations. First, figural stimuli may be approximations since they are based on estimated body shapes, which may lead to respondents’ misperceptions [21–23]. Second, figural stimuli are not developed from objective anthropometric measures, thus limiting their use for comparing body shape perception to anthropometry-related health outcomes [24–26].

To overcome these limitations in African populations, two photographic scales based on real African phenotypes were created: the Body Size Scale (BSS) and the Body Image Scale (BIS) [27, 28]. Although these scales were developed to cover the variability of adiposity in African populations, they only express a continuum in body weight variation. Other anthropometric traits of body shape including musculature, belly, buttock and breast shapes are not included in these scales.

Body shape scales based on real human bodies have been developed to study sexual attractiveness, but these have been designed from European phenotypes [29–31]. Several studies have used scales designed for Europeans in African populations [32–34]. The Somatomorphic Matrix-male is a bi-dimensional computerized body image assessment tool based on real human bodies to assess body shape perceptions in males [35], and recently upgraded to assess reliable actual-ideal body discrepancy [36]. In spite of this, the tool is also phenotypically Eurocentric; its construction is based on two anthropometric dimensions expressing body shape: fat-free mass (muscular component) and body fat (fatness component). Our study builds on this formative research through the development and validation of the Body Shape Scale (BOSHAS), a photographic tool integrating three body shape components: muscularity, fatness and slimness, designed specifically for assessing body image in Africans. Based on the somatotyping method developed by Heath and Carter [37], an anthropometric technique integrating these three body shape dimensions simultaneously, the BOSHAS is an innovative sex-specific photographic scale of real African models that captures varying African body shape phenotypes. Therefore, this study aims to develop and validate the BOSHAS to evaluate body shape perceptions related body image disorders in African populations.

## Methods

### Development of the body shape scale

#### Populations

Subjects were recruited in the Niger–Congo region, Africa’s largest ethno-linguistic area spreading from Western to Southern Africa. Within this wide area, Bantoid (Western African ancestry group) and Bantu (Central African ancestry group) populations were selected, since they are known as linguistically distinct [38], phenotypically adapted to their contrasted ecological environment (lanky Western Bantoid in the Sahel vs. stocky Bantu in the equatorial forest) [39], and genetically different [40]. Bantoid and Bantu subjects were selected in Senegal and Cameroon, respectively [37].

#### Sampling

The sample included 161 Senegalese and Cameroonian adult subjects (age range: 18–75 years), 31 males and 50 females from Dakar (Pikine area), and 51 males and 29 females from Yaoundé (Cité Verte area). The participants of the BOSHAS development protocol were recruited as part of a wider health survey to estimate the prevalence of obesity, diabetes and hypertension in both regions. Participants who were pregnant, < 18 years old or with a collapsed body posture from old age, were not recruited for this study because the aim was to collect photographs of adult bodies for the BOSHAS that did not belong to any specific age range within adulthood.

#### Photographs

A dedicated room was rented in both cities during data collection. A Canon EOS 450D camera (Tokyo, Japan) was set up on a tripod height of 1 m, using an 18–55 mm lens. Using the maximum resolution of the camera (4272 × 2848 pixels), the front and the left side of participants were photographed. More technical details concerning the use of the camera and the position of participants are available in the development and validation of the BSS [27]. The mediolateral and the anteroposterior body axes were of particular interest to depict the variation in body shape. Indeed, this latter axis is rarely represented in existing body scales, even though its potential contribution to body image assessment studies [41, 42].

#### Anthropometric measurements

Subjects were measured using the body mass index (BMI), the body fat distribution and the somatotype standardized procedures to evaluate their morphology accurately. We used WHO BMI cut-offs to define underweight (< 18.5 kg/m^2^), normal-weight (18.5–24.9 kg/m^2^), overweight (≥25 kg/m^2^) and obesity (≥30 kg/m^2^) of subjects. Since this international BMI classification is not fully adapted to body composition variations across ethnic-groups, as in African populations [43], we complemented the BMI measurement with measures of body shape including body fat distribution and composition, as recommended by Adab et al. [44].

A digital beam scale (Tanita, Tokyo, Japan) was used to measured weight to the nearest 0.1 kg. A portable stadiometer (Siber-Hegner, Zurich, Switzerland) was used to measure standing height to the nearest mm. BMI (in kg/m^2^) was calculated by dividing weight (in kg) by squared height (in m). Hip, waist, upper arm (flexed and tensed) and calf circumferences were measured to the nearest mm in a standing position using a tape measure [45]. Waist-to-hip ratio (WHR) was calculated. A Harpenden skinfold caliper (Holtain Ltd., Crymych, UK) was used to measure triceps, supraspinal, subscapular and medial calf skinfold thicknesses to the nearest mm [46]. A Mitutoyo Dial Caliper (Mitutoyo America, Aurora, Illinois, USA) was used to measure the biepicondylar humerus and femur bone breadths.

Using the Somatotype 1.0 software package (MER Goulding Software Development, Geeveston, Australia), the somatotype of each participant was assessed from the calculation of its ternary component (musculature, slimness and fatness indexes) based on ten of the anthropometric measures described above [37], and stated in the BSS development protocol [27]. The somatocharts showing the somatotype distribution in our sample were also generated using this software. The assessment of the somatotype category was defined by combining the respective degrees of fatness (endomorphy), muscularity (mesomorphy) and slimness (ectomorphy) interpreted by a unit system from the Heath and Carter somatotyping metric [37]. The ternary components of each somatotype were plotted to represent subjects’ indices of musculature, slimness and fatness, respectively.

#### Selection of the models

Ten subjects per sex were selected as models for the BOSHAS; this number provides an optimal trade-off between having a large enough choice with sufficient reliability [47]. We carefully ensured that we encompassed the anthropometric variability from the study population in terms of body shape (somatotype), adiposity (BMI) and body fat distribution (WHR). In our study population, we considered these three inclusion criteria simultaneously to select BOSHAS models. First, we randomly selected multiple clusters (i.e. small subsamples) within each of the main somatotype categories, as weakly endomorph, moderately mesomorph, strongly ectomorph. Second, we randomly chose a few individuals with the most contrasted BMI values from these clusters; and third, we retained individuals (the models) with the most opposite WHR for the BOSHAS within these last smaller clusters. Through this triangulation between somatotype, BMI and WHR, we retained different degrees of endomorph, mesomorph and ectomorph models for the BOSHAS, with both BMI and WHR variability, as explained below.

Based on the somatotyping metric, we selected five endomorph subjects for the female scale: one normal-weight (WHR: 0.86) strongly endomorph (“Endo++”: endomorphy is 0.5 unit higher than the two other components), two overweight (WHR: 0.86 and 0.95) and one obese (WHR: 0.80) moderately endomorph (“Endo+”: endomorphy is the first component but the second one is 0.5 unit higher than the third one) and one normal-weight (WHR: 0.68) weakly endomorph (“Endo-”: endomorphy is the first component but within a 0.5-unit range from the second component). We then selected one underweight (WHR: 0.75) relatively ectomorph subject (Ecto+) and one normal-weight (WHR: 0.82) central subject (all components are within a 1-unit range from each other). Finally, we selected three mesomorph subjects: one normal-weight (WHR: 0.78) strongly mesomorph (Meso++), one overweight (WHR: 0.74) and one obese (WHR: 0.84) relatively mesomorph (Meso+).

For the male scale, we selected two endomorph models: one overweight (WHR: 0.85) relatively endomorph (Endo+) and one overweight (WHR: 0.93) weakly endomorph (Endo-), one underweight (WHR: 0.81) strongly ectomorph (Ecto++), one normal-weight (WHR: 0.82) central subject, and six mesomorph subjects: one normal-weight (WHR: 0.87) strongly mesomorph (Meso++) and five relatively mesomorph (Meso+), one normal-weight (WHR: 0.88), one overweight (WHR: 0.97) and three obese (WHR: 0.84, 0.94, 0.98).

Adobe Photoshop CS software (Adobe Systems Inc., San Jose, CA) was used to edit the photographs, mask the faces, remove the shadows and introduce a white background.

### Validation of the body shape scale

#### Population

A validation protocol was conducted in a random sample of Senegalese adults from Dakar (urban) and Kaolack (rural). In every 3rd household, male and female adults informed and aware of the study procedure and providing their agreement were invited to participate in the study.

#### Administering the BOSHAS

Although the BOSHAS presents different human body shapes, these models are not arranged as decreasing or increasing body shape metrics. However, it is possible to validate this tool objectively according to the body shape preference of participants. For instance, participants pointed out different models for several body shape criteria (e.g. preference order, Current Body Shape (CBS)) at two different time points. This allowed us to determine the repeatability (test-retest) of their perceptions objectively. Hence the photographs were presented on large individual cards (width: 10 cm, length: 20 cm) so that the participants could accurately observe the body shape differences between models. The photos were also randomly numbered from 1 to 10 (the numbers were blinded on the back of the cards), shuffled at every question and presented to the participants blinded from any anthropometric information [47].

#### Test-retest reliability

We assumed that the preference order of the models in the BOSHAS stated by participants constitutes a relevant dimension that could be used to evaluate the validity of this body image scale. Hence, we assessed the repeatability of the participants’ order to evaluate whether the BOSHAS allowed them to find their own hierarchical preference of the models. Accordingly, we asked the participants twice, with a test-retest interval of 2 weeks, to rank the BOSHAS models according to their preference order, a strategy of validation based on the classification of the models already applied for the BIS [28], the Body Size Guide (BSG) [48] and the Photographic Figure Rating Scale (PFRS) [49]. We recruited 106 Senegalese adults of both sexes (45 females and 61 males; age range: 18–77 years) who expressed their preference order on the BOSHAS for themselves as well as for their partner (for polygamous households, the wife was chosen by the husband). For own sex perception assessment, males were administered with the male scale and females with the female scale. For the partner perception assessment, the male and female scales were reversed. For both of the sex-specific subscales, we asked the following question in French or Wolof: *“Peux-tu classer ces images de celle que tu préfères le plus à celle que tu préfères le moins*? *”* [Can you sort these models from your most to least preferred?]

As carried out for the PFRS and the BSS [27, 49], we administered the BOSHAS in the same sample using the protocol described earlier to evaluate the reliability of assessing current body shape (CBS and CBS’), desired body shape (DBS and DBS’) and ideal body shape (IBS and IBS’) for oneself and one’s partner. We evaluated the participant’s CBS by asking *“Peux-tu montrer l’image qui te ressemble le plus?*” [Can you point out the model that looks the most like you?]. We evaluated DBS by asking *“Peux-tu montrer l’image à laquelle tu veux ressembler le plus?”* [Can you point out the model that you most want to look like?]. We evaluated IBS by asking *“Peux-tu montrer l’image idéale pour toi?”* [Can you point out the ideal model for you?]. The difference between the DBS and IBS consists of the DBS means the reachable body shape expected by the participant and the IBS means the ideal body shape in absolute terms without the personal individual expectation [50]. We repeated the same protocol to assess perceptions of their partner’s body shape. We evaluated CBS’ by asking *“Peux-tu montrer l’image qui ressemble le plus à ton partenaire?”* [Can you point out the model that looks the most like your partner?]. We evaluated DBS’ by asking *“Peux-tu montrer l’image à laquelle tu veux que ton partenaire ressemble le plus?”* [Can you point out the model that you most want your partner to look like?]. We evaluated IBS’ by asking *“Peux-tu montrer l’image de partenaire idéal pour toi?”* [Can you point out the ideal partner model in your opinion?]. CBS/CBS’, DBS/DBS’ and IBS/IBS’ scores were defined as the number corresponding to the photo that the participant selected.

#### Convergent validity

We recruited 597 adult Senegalese (age range: 18–101 years) living in Dakar agglomeration and Kaolack region (284 males and 313 females). The aim was to compare body shape perceptions assessed with the BOSHAS with those obtained by an interview-administered questionnaire, as examined in previous studies to test the convergent validity of the PFRS and the BSS [27, 51]. Usually, the methodological strategy consists of correlating dimensions assessed by a body scale (e.g. body dissatisfaction) with existing validated questionnaires measuring similar dimensions. However, our knowledge of the literature shows that no culturally relevant questionnaire on body image had been developed for African populations, and no body image questionnaire had been culturally adapted to African populations. The Body Shape Questionnaire might be an appropriate tool to be correlated with a body shape scale [50]. However, the cross-cultural adaptation of existing questionnaires is a complicated process [52], especially in the African context where large body shapes are still valued and food is perceived as a symbol of wealth [53], while body image questionnaires used in HIC populations tend to integrate slimness valorisation and eating disorders as norms. Traditional African aesthetics are not based on the rejection of the body and flesh, and individuals are still relatively protected from advertising and peer pressure to constantly improve their appearance, as observed in a recent qualitative socio-anthropological study in Senegal [54]. Therefore, we decided to develop an original culturally relevant questionnaire for this study (Table S2), based on the main findings of the previous socio-anthropological study, to specifically assess the convergent validity of the BOSHAS. We were able to create a simple questionnaire adapted to Senegalese body shape perceptions, which was recommended as an important criterion concerning the cross-cultural adaptation of existing questionnaires [55]. Accordingly, the following questions were administered by questionnaire:

- Body weight self-satisfaction (variable 1)

*“Sur un plan esthétique, vis-à-vis de votre poids, vous vous trouvez : 1. trop maigre, 2. un peu trop maigre, 3. normal, 4. un peu trop gros, ou 5. trop gros?”* [“With regards to appearance, concerning your weight, do you feel: 1. too slim, 2. a bit too slim, 3. normal, 4. a bit too fat or 5. too fat?”].

For this item, we wanted to compare body weight self-satisfaction assessed by this question with that assessed by the BOSHAS.

- Body self-assessment (variable 2)

*“Quelle silhouette avez-vous selon vous : lutteur, charpenté, ventru, musclé, « sec » (pour les hommes), « taille-fine », « taille Coca-Cola », enrobée, fessue, forte (pour les femmes), moyen, mince, décharné, menu, gros, autre (pour les deux sexes)?”* [“Which silhouette are you: wrestler, sturdy, potty, muscular, “muscular and thin” (for men), “slim-waist”, “Coca-Cola body”, coated, large-buttocks, sharp (for women), medium, slim, skinny, menu, big, other (for both)”].

For this item, we used common vernacular Wolof terms to qualify silhouettes, here translated into French and English. These terms were identified in a recent socio-anthropological study conducted in Senegal [54]. We compared the responses to this question to the BOSHAS-assessed CBS.

- Relationship with appearance (variable 3)

*“Sur un plan esthétique, quelle place accordez-vous à votre apparence physique (silhouette, poids, traits, etc…) : 1. aucune, 2. très modérée, 3. modérée, 4. relativement importante, 5. importante?”* [“With regards to aesthetics, what importance do you give to your appearance: 1. none, 2. slightly important, 3. moderate, 4. relatively important or 5. important?”].

- Aesthetics (variable 4)

*“Vous voudriez être dans la norme de beauté moderne occidentale ou votre apparence physique importe peu?”* [“Would you like to meet Western modern body norms or is your physical appearance not important for you?”].

We asked these two last questions because a socio-anthropological study [54] in Senegal shows that ‘Western’ acculturation through media promotes an increasing importance of physical appearance, not valued by traditional rural society, where one’s generosity and vitality are the most valued facets. We compared the responses from these items to the DBS, IBS and body self-satisfaction assessed by the BOSHAS.

We also compared the results from the BOSHAS to objective anthropometric measures [56]. We measured participants’ weight and height and we derived BMI classification to compare these anthropometric parameters to CBS (concurrent validity).

### Statistics

#### Sociodemographic and somatotype analysis

The characteristics of the three samples were described with means (± Standard Deviation, SD) and percentages. The somatotype analysis provided the somatotype profile of each subject. Frequency comparisons were then carried out to test the differences in somatotype status with regard to the country of origin, by using Fisher’s exact test.

#### Reliability

For each sex-specific subscale, we calculated simple and weighted Cohen’s Kappas to compare the agreement between the first and second preference order of the participants. Test-retest reliability of CBS, DBS and IBS were evaluated using Spearman’s correlations and simple Cohen’s Kappas. We did not use weighted kappas, since the BOSHAS was not designed for use in a specific ascending/descending order.

#### Convergent validity

We assessed concurrent validity of both sex-specific subscales by estimating the Spearman’s correlation between CBS (categorized by ascending BMI) and objective BMI as continuous. We also categorized BMI and CBS into four categories (underweight, normal weight, overweight and obesity) and evaluated their agreement using simple and weighted Cohen’s Kappas of both subscales.

We derived a body self-satisfaction variable from the BOSHAS: *satisfied* participants were those who pointed out the same silhouette for CBS and DBS, while the others were considered *not satisfied* [57]. We compared the BOSHAS-derived *self-satisfaction* to the *body weight self-satisfaction* as declared by questionnaire (variable 1: normal vs. other modalities) through the sensitivity and specificity estimations as in previous studies [58–60].

We derived a “normal” body self-assessment variable from the BOSHAS corresponding to all silhouettes as perceived CBS valued in Senegalese society: ‘Coca-Cola’ body (model 5: a thin women with large buttocks and big breasts) and medium body (models 2, 3 and 5) in women, and wrestler and muscular (model 4, 7 and 10) and medium (model 1, 4, 7 and 8) bodies in men. A medium body on both scales corresponds to models between normal-weight bodies that are a bit fleshy, and overweight bodies without a big belly, for both sexes. These morphologies (1, 4, 7, 8 for men; 2, 3, 5 for women) represent vitality for the Senegalese beyond recent differences noted between perceptions based on age and associated with ‘Western’ acculturation [54]. On BOSHAS, we coded all models that corresponded to these silhouettes as *normal CBS*. We tested whether *normal CBS* predicted the self-assessed *normal body* on the questionnaire (variable 2: “normal” silhouettes as wrestler, Coca-Cola body, medium vs. others) by estimating sensitivity and specificity. We also tested whether BOSHAS-derived *self-satisfaction* predicted the *normal body* (variable 2).

Finally, we derived *modern DBS/IBS* variables from BOSHAS. *Modern DBS/IBS* corresponded to models 4 and 10 on the male scale and 3 and 5 on the female scale. Indeed, young people (possibly influenced by ‘Western’ media) value muscular bodies in men, and “sexy” bodies in women, defined as having large buttocks and breasts, but no fat. The value of these exigent aesthetic criteria could lead to body dissatisfaction [8]. Therefore, we tested whether BOSHAS-derived *self-dissatisfaction* predicted the *importance of appearance* (variable 3: relative/important vs. other modalities). Similarly, we tested whether the BOSHAS-derived *modern DBS/IBS* and *self-dissatisfaction* predicted *modern aesthetic criteria* (variable 4: modern vs. no modern).

Statistical analyses were performed using STATA 12 (StataCorp, College Station, TX, USA).

## Results

### Development of the body shape scale

#### Anthropometric characteristics

Selected Cameroonian males were more likely to be mesomorph and Senegalese males more likely to be ectomorph; frequency of endomorphy was similarly low in both groups (*p* < 0.01; Table S1). Similar, but less pronounced trends in ectomorphy and mesomorphy were found between Cameroonian and Senegalese females; however, in contrast to males, the frequency of endomorphy was similarly high in both groups (*p* < 0.05).

#### The body shape scale

**Fig. 1:**
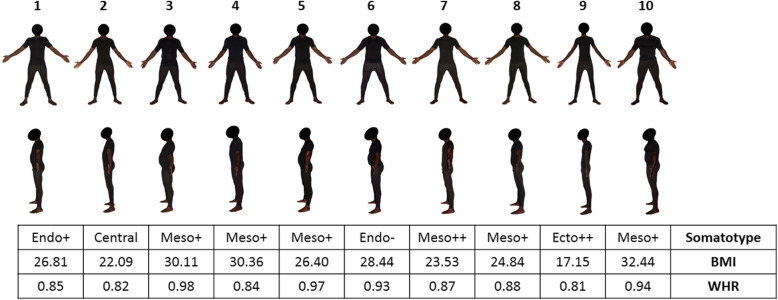
Male Body Shape Scale. The scale shows 10 Black male models from front and left side-views. The table gives the somatotype component, the body mass index and the waist-to-hip ratio corresponding to each model. BMI: Body Mass Index. WHR: Waist-to-Hip Ratio

**Fig. 2:**
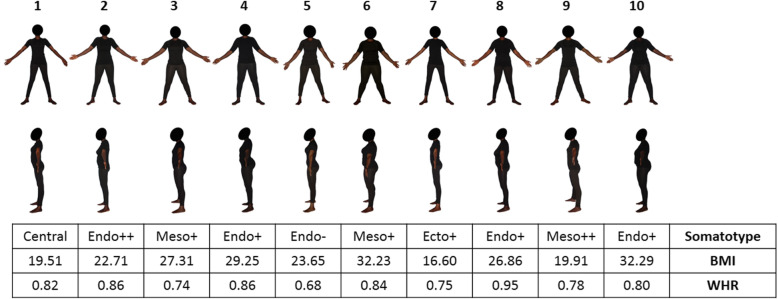
Female Body Shape Scale. The scale shows 10 Black female models from front and left side-views. The table gives the somatotype component, the body mass index and the waist-to-hip ratio corresponding to each model. BMI: Body Mass Index. WHR: Waist-to-Hip Ratio

**Fig. 3:**
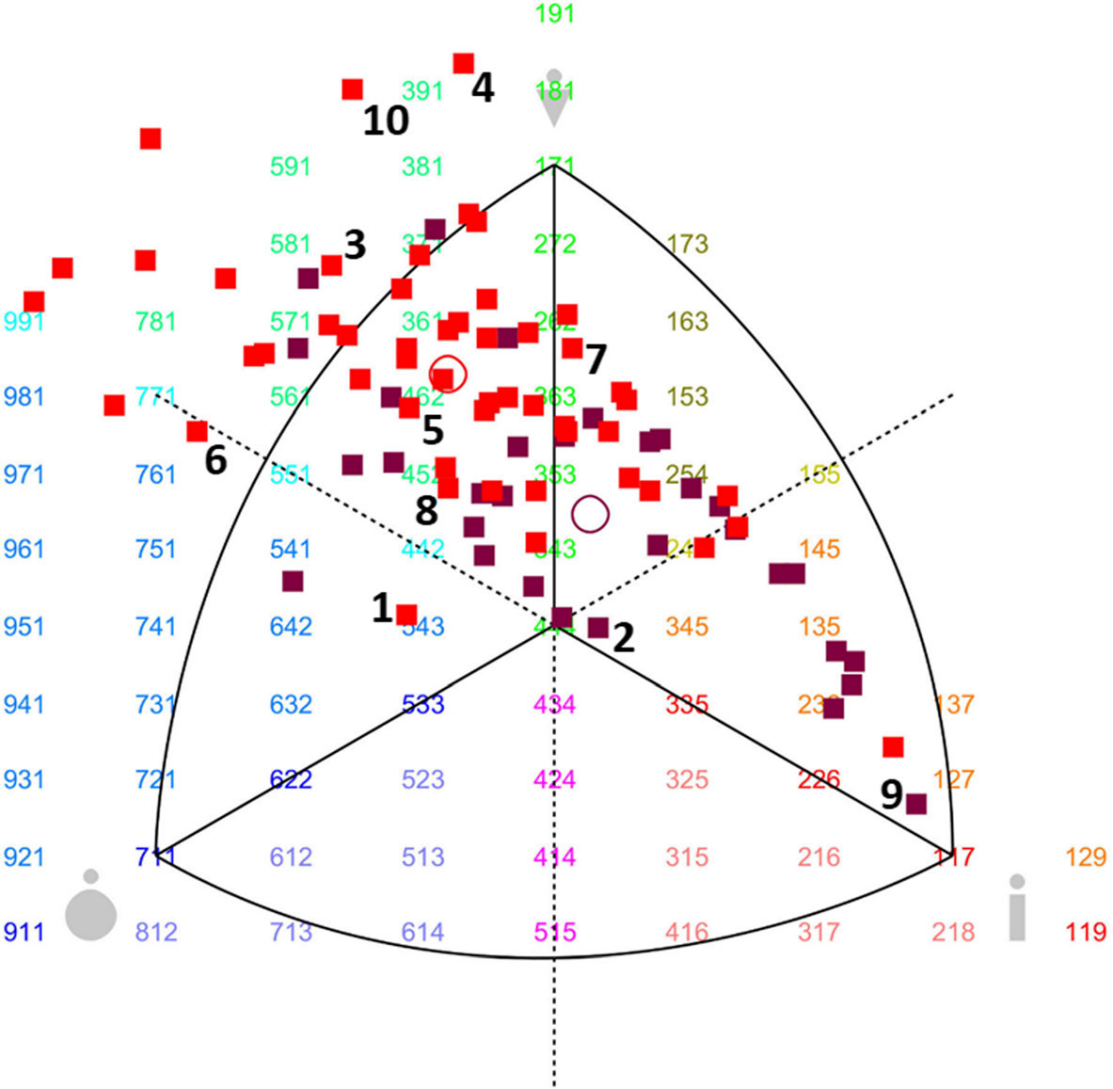
Somatotype profiles of male candidates as models for the BOSHAS. In somatocharts (generated with the Somatotype 1.0 software package), mesomorphy is represented by the vertical axis pointing upward; endomorphy by the horizontal axis pointing to the left; and ectomorphy by the horizontal axis pointing to the right. The coordinates 951 on the left side present, for instance, the highest degree of endomorphy [9], a medium degree of mesomorphy [5] and the lowest degree of ectomorphy [1]. The red squares represent Cameroonian males and the purple ones Senegalese males. The red circle indicates the mean profile for Cameroonians and the purple one the mean profile for Senegalese. All squares encircled represent models selected for the BOSHAS

**Fig. 4:**
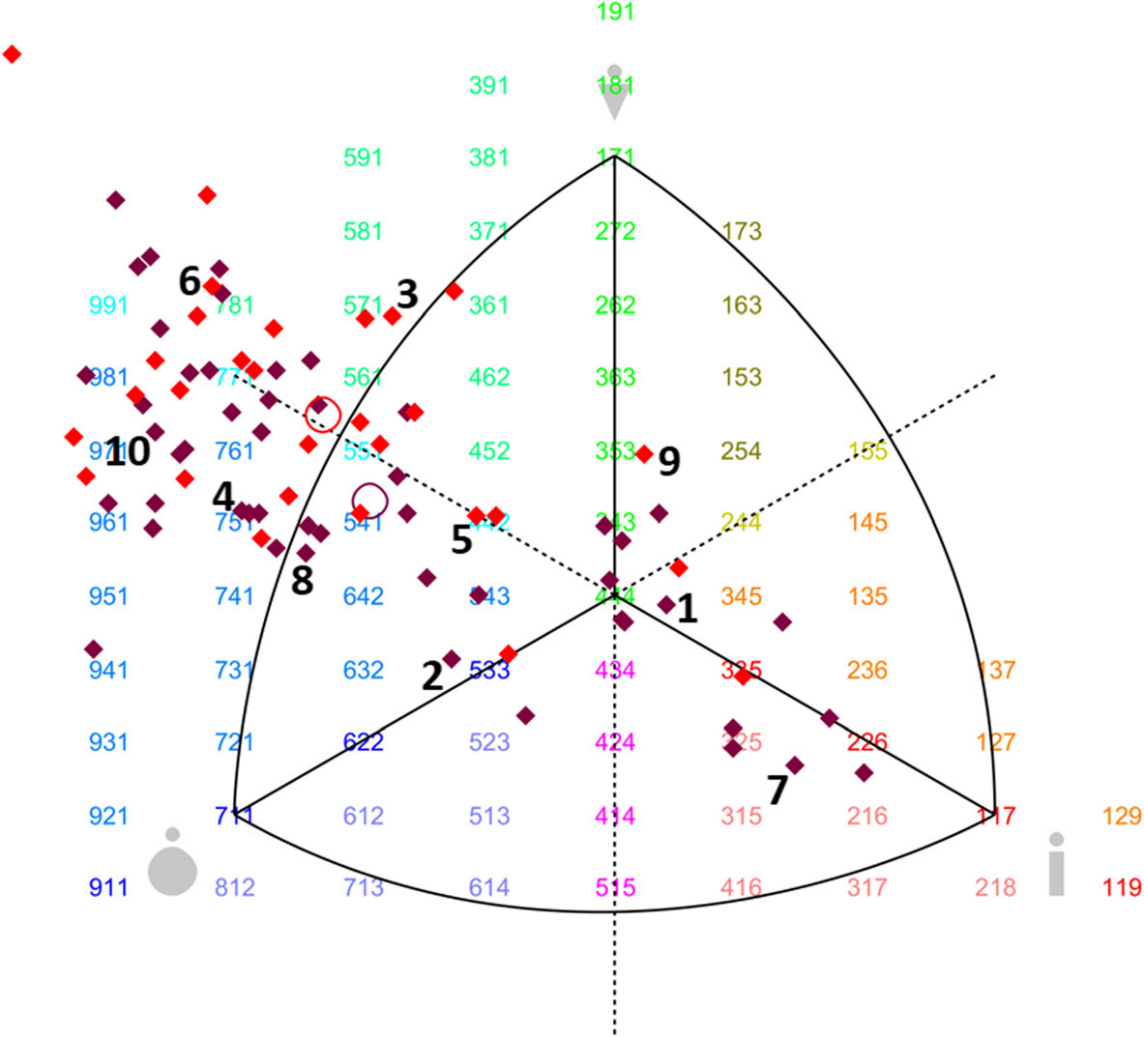
Somatotype profiles of female candidates as models for the BOSHAS. In somatocharts (generated with the Somatotype 1.0 software package), mesomorphy is represented by the vertical axis pointing upward; endomorphy by the horizontal axis pointing to the left; and ectomorphy by the horizontal axis pointing to the right. The coordinates 951 on the left side present, for instance, the highest degree of endomorphy [9], a medium degree of mesomorphy [5] and the lowest degree of ectomorphy [1]. The red diamonds represent Cameroonian females and the purple ones Senegalese females. The red circle indicates the mean profile of Cameroonians and the purple one the mean profile of Senegalese. All diamonds encircled represent models selected for the BOSHAS

Figures 1 and 2 show the sex-specific subscales of real African models photographed from both face and profile, containing the 10 males/10 females selected as models for the BOSHAS, together with their corresponding anthropometric data. The models selected are not arranged in any specific order based on anthropometric characteristics but cover the somatotype variability of the initial sample. The wide variability of somatotype components in the BOSHAS for each sex is presented in the two somatocharts as Figs. 3 and 4. Secondly, the BOSHAS also integrated BMI and WHR variability within somatotype variability.

### Validation of the body shape scale

Senegalese participants were enrolled to assess test-retest reliability (*n* = 106) and convergent validity (*n* = 597). Descriptive characteristics of these two independent samples are described in Table 1.

**Table 1:** Descriptive characteristics of the population samples enrolled for the Body Shape Scale validation protocol

	Test-retest reliability	***SD***	Convergent validity	***SD***
Sample size, *n*	106		597	
Age, *y*	31.2	*12.6*	36.7	*15.6*
Sex, *% males/females*	58.0 / 42.0		47.6 / 52.4	

*SD* Standard Deviation

Table 2 presents the test-retest reliability of the preference order of the 106 participants. Overall, participants were consistent in ranking the models by their preference order on both male and female scales 2 weeks apart (kappa coefficients ranging from 0.50 to 0.72). Younger people (< 30 years) and males exhibited a higher consistency than older people (≥30 years) and females, respectively.

**Table 2:** κ and weighted κ corresponding to the test-retest reliability of the Body Shape Scale to assess the preference order, according to age and sex of the participants

	Preference order for oneself and partner			
	< 30 years	≥30 years	Male	Female
**Male scale**				
*Κ*	0.69	0.58	0.69	0.61
*weighted κ*	0.68	0.63	0.70	0.63
**Female scale**				
*Κ*	0.71	0.52	0.64	0.63
*weighted κ*	0.72	0.50	0.67	0.61

Test-retest reliability performances of CBS, DBS and IBS for oneself and partner on both sex-specific subscales are shown in Table 3. In both subscales, the correlation coefficients were found to be significant and the kappa coefficients were between moderate and good, except for the DBS/DBS’ on both scales for which the agreement was fair (between the first and second time tested). The agreements between these three variables were also all significant, indicating a strong consistency for assessing different body image dimensions within a 2 week interval.

**Table 3:** Spearman’s correlation and Cohen’s simple corresponding to the test-retest reliability of the Body Shape Scale to assess current, desired and ideal body size of participants

	***Ρ***	***Κ***
**Male scale**		
CBS/CBS’ (*n* = 102)	0.52***	0.49
DBS/DBS’ (*n* = 103)	0.40***	0.37
IBS/IBS’ (*n* = 106)	0.79***	0.84
**Female scale**		
CBS/CBS’ (*n* = 99)	0.77***	0.79
DBS/DBS’ (*n* = 105)	0.24***	0.28
IBS/IBS’ (*n* = 106)	0.71***	0.80

*CBS* Current Body Shape, *DBS* Desired Body Shape, *IBS* Ideal Body Shape

*CBS’* Current Body Shape, *DBS’* Desired Body Shape, *IBS’* Ideal Body Shape: for partner

*P* values of Spearman’s correlations: * < 0.05; ** < 0.01, *** < 0.001

Concurrent validity was assessed by calculating the correlation between CBS and BMI. Spearman’s coefficients were 0.42 for males and 0.66 for females (both *p* < 0.001). Simple and weighted Kappa coefficients between BMI and CBS categories in males were respectively 0.08 and 0.24, i.e. poor to fair significant agreement between the two variables. In females, weighted Kappa coefficients between BMI and CBS categories were respectively 0.21 and 0.56, i.e. fair to moderate significant agreement between the two variables.

Table 4 summarises the sensitivity and specificity of sex-specific BOSHAS subscales regarding the prediction of body weight self-satisfaction, self-assessed normal body, importance of appearance and modern aesthetic criteria, assessed by the questionnaire (*n* = 597 Senegalese). As an example, 70% of participants who declared by questionnaire that they were satisfied with their body weight were correctly identified by the BOSHAS as satisfied with their body (sensitivity). Specificity was slightly higher, as 71% of participants categorised as unsatisfied with their body from the questionnaire were correctly identified as unsatisfied by the BOSHAS. Sensitivity and specificity ranged from 52 to 72%.

**Table 4:** Values of sensitivity and specificity of the Body Shape Scale to identify body weight self-satisfaction, normal body self-assessment, importance of appearance and modern aesthetic criteria in the overall sample

	Sensitivity	Specificity
**Body weight self-satisfaction**		
Self-satisfaction	70%	71%
**Normal body**		
Normal CBS	70%	52%
Self-satisfaction	72%	53%
**Importance of appearance**		
Modern DBS	59%	55%
Modern IBS	54%	54%
Self-dissatisfaction	52%	54%
**Modern aesthetic criteria**		
Modern DBS	63%	53%
Modern IBS	63%	54%
Self-dissatisfaction	58%	54%

*CBS* Current Body Shape, *DBS* Desired Body Shape, *IBS* Ideal Body Shape

## Discussion

The main aim of this study was to develop and validate the Body Shape Scale (BOSHAS) to assess body shape perceptions in African populations. This tool is innovative because it contains two sex-specific subscales of 10 photographs of real Black African models with a wide range of body shapes. It is based on three combined anthropometric criteria: the somatotype, BMI and WHR. A validation protocol confirmed the validity and reliability of the BOSHAS for the Senegalese adult population.

The models used in the BOSHAS were selected from a sample encompassing a large anthropometric variability in terms of body shape within Senegalese and Cameroonian populations. We observed that the Senegalese tended to be more ectomorph, whilst Cameroonians tended to be more mesomorph. These results are in accordance with findings from recent studies conducted in Senegal and Cameroon [61, 62], as Cameroonians have a higher BMI than Senegalese, which impacts on the somatotype frequency [63]. The BOSHAS is the first body image scale to rigorously and accurately capture a large variation in African body shape.

First, real African models of both genders standing in two positional views were captured by the BOSHAS, so this tool represents a significant improvement in the development of body image scales for Africans [41]. Indeed, the BOSHAS avoids misestimating that results from using silhouettes or drawings [20, 23], or inappropriate Eurocentric photographic body image scales, ill-adapted to African populations [29, 30]. In addition, the BOSHAS adds to a body of evidence, which has limited anthropometric data on body shapes scales for both males and females [22, 23, 49, 64], and seldom incorporates frontal and side views [22, 23, 65]. The side view represents the belly, buttocks and breast shapes best [41], which are criteria contributing to body attractiveness [31, 66, 67]. Whilst computerized methodologies are available to capture body shape in three-dimensions, they do not present real human models, are expensive, and remain unfeasible for large scale epidemiological studies in low and middle income countries [29, 68–70]. Additionally, the BOSHAS captured varying African body shape phenotypes by presenting 10 models on each sex-specific subscale including somatotype (i.e. different degrees of mesomorphy, ectomorphy and endomorphy simultaneously), BMI and WHR, as multiple anthropometrical dimensions of body shape, while the Somatomorphic Matrix-Male included two anthropometrical dimensions [35, 36].

This validation protocol demonstrated that the use of the BOSHAS in the Senegalese population is reliable. First, the aptitude of participants to consistently arrange the models according to their preference order for themselves and their partner 2 weeks apart was moderate to good. Second, the test-retest reliability performed 2 weeks apart for themselves and their partner was good. Third, the concurrent validity between CBS and BMI was good in both sexes. Fourth, the assessment of convergent validity showed relatively high predictive values for participants’ *body weight self-satisfaction, normal body, importance of appearance and modern aesthetic criteria*.

The main limitation of the BOSHAS is that it was only validated in the Senegalese population. Even if body scales have been used with populations different to those that they were originally developed and validated for [33, 34], the BOSHAS would benefit from further validation in other African populations, living in Africa or elsewhere. The second limitation is that the concurrent validity of the BOSHAS was slightly weak based on kappas and weighted kappas. A possible explanation could be that the BOSHAS expresses a body shape variation whereas BMI expresses a body size variation. Future studies could correlate CBS with the somatotype of participants, but for this present study it was unfeasible to assess the somatotype of 597 subjects in the field.

Despite these relatively low kappa coefficients, agreements between CBS and BMI were demonstrated and significant in both subscales. Globally, the validation of the BOSHAS presented reliable performances similar to other existing body image scales synthesised in the literature [56]. Our innovative body image assessment tool can be used in African populations, as other relevant photographic scales (PFRS/BSG) assessing body image have been in ‘Western’ populations [48, 49]. The BOSHAS contributes to existing methods of assessing body image by providing a novel body scale based on real and variable African body phenotypes to specifically and rigorously assess body shape perceptions in African populations. In the context of a globalised consumer driven society, accompanied by a media-driven portrayal of the *perfect body* [12], the BOSHAS can be used to estimate the extent to which the social value of *modern bodies* represents a risk factor for dysmorphic or eating disorders in African populations.

## Conclusions

The BOSHAS is a unique, innovative and reliable tool for assessing body shape perceptions in African populations. It allows the measurement of associations between body shape perceptions, body shape dissatisfaction and potential body dysmorphic and eating disorders. The BOSHAS can therefore detect and help in the prevention of these body image disorders in African populations. It may also be used in comparative cross-cultural studies to assess differences in body shape perception and related body image disorders in native Africans and African diaspora.

## Supplementary information

**Additional file 1: Table S1.** Frequencies of somatotype components in Cameroonians and Senegalese. This table presents the somatotype profiles in the sample used for the development of the BOSHAS.

**Additional file 2: Table S2.** Original culturally relevant questionnaire for the convergent validity of the BOSHAS.

## Data Availability

The datasets used and/or analysed during the current study are available from the corresponding author on reasonable request (except the photographs).

## Abbreviations

BIS: Body Image Scale
BOSHAS: Body Shape Scale
BMI: Body Mass Index
BSG: Body Size Guide
BSS: Body Size Scale
CBS: Current Body Size
DBS: Desired Body Size
Ecto: Ectomorphy
Endo: Endomorphy
HIC: High-Income Countries
IBS: Ideal Body Size
Meso: Mesomorphy
PFRS: Photographic Figure Rating Scale
